# Clinical Characteristics of Vitamin D Deficiency Detected in Long COVID Patients During the Omicron Phase

**DOI:** 10.3390/nu17101692

**Published:** 2025-05-16

**Authors:** Yui Matsuda, Yasue Sakurada, Yasuhiro Nakano, Yuki Otsuka, Kazuki Tokumasu, Hiroyuki Honda, Yoshiaki Soejima, Yuya Yokota, Ryosuke Takase, Daisuke Omura, Fumio Otsuka

**Affiliations:** Department of General Medicine, Okayama University Graduate School of Medicine, Dentistry and Pharmaceutical Sciences, Okayama 700-8558, Japan; phvw0350@okayama-u.ac.jp (Y.M.); pzaf6h9w@s.okayama-u.ac.jp (Y.S.); y-nakano@okayama-u.ac.jp (Y.N.); otsuka@s.okayama-u.ac.jp (Y.O.); tokumasu@okayama-u.ac.jp (K.T.); ppgf1hrd@okayama-u.ac.jp (H.H.); p32v0ja8@s.okayama-u.ac.jp (Y.S.); psu62qc4@okayama-u.ac.jp (Y.Y.); p4v05asb@okayama-u.ac.jp (R.T.); me20011@s.okayama-u.ac.jp (D.O.)

**Keywords:** COVID-19, 25-hydroxyvitamin D, long COVID, palpitation, vitamin D deficiency

## Abstract

**Background:** To characterize the clinical significance of vitamin D deficiency (VDD) detected in long COVID, a retrospective observational study was performed for outpatients who visited our clinic during the period from May 2024 to November 2024. **Methods:** Clinical trends in long COVID patients diagnosed with VDD who showed serum concentrations of 25-hydroxyvitamin D (25-OHD) lower than 20 ng/mL were compared with those in long COVID patients in a non-deficient vitamin D (NDD) group. **Results:** Of 126 patients with long COVID, 97 patients (female: 50) who had been infected during the Omicron phase were included. Sixty-six patients (68%) were classified in the VDD group. The median serum concentrations of 25-OHD were 14.8 ng/mL in the VDD group and 22.9 ng/mL in the NDD group. There were no significant differences between the two groups in terms of age, gender, BMI, severity of COVID-19, period after infection and vaccination history. Although the levels of serum calcium and phosphate were not significantly different between the two groups, the percentages of patients in the VDD group who complained of dizziness, memory impairment, palpitation and appetite loss were larger than those in the NDD group. Of note, the patients who complained of palpitation showed significantly lower concentrations of serum 25-OHD than those in the patients without palpitation (median: 11.9 vs. 17.3 ng/mL). Moreover, patients in the VDD group had significantly higher scores for physical and mental fatigue as well as higher scores for depressive symptoms. **Conclusions:** Collectively, VDD is involved in clinical manifestations of long COVID, particularly symptoms of palpitation, fatigue and depression.

## 1. Introduction

Since the declaration of a global pandemic of coronavirus disease 2019 (COVID-19) caused by severe acute respiratory syndrome coronavirus 2 (SARS-CoV-2), COVID-19 has caused acute symptoms as well as a prolonged post-COVID-19 condition (PCC) or long COVID sequelae [1]. Prolonged conditions and related symptoms after COVID-19 have been reported to affect up to 20% of COVID-19 patients as early as two months after COVID-19 onset, and symptoms include fatigue, headache, insomnia, dyspnea and dysgeusia, memory and cognitive impairment, hair loss, and so on [2,3,4,5]. Although the specific pathogenesis and treatment for long COVID have not yet been established, several endocrine factors, including hypothalamo-pituitary hormones, adrenocortical hormones and sex steroids, have been suggested to be involved in the development of long COVID [6,7,8,9,10].

It has been reported that micronutrients such as vitamin D and zinc play a key role in the body’s response against oxidation, inflammation and thrombosis and that the micronutrients are useful for immuno-modulation and -adaptation [11]. Vitamin D depletion seems to increase the risk, severity, morbidity and mortality of respiratory diseases and viral respiratory infections, including COVID-19 [12], whereas zinc deficiency was detected in 22% of the Japanese long COVID patients in our previous study [13].

As for the significance of vitamin D in patients with long COVID, the involvement of vitamin D depletion in long COVID in 100 COVID-19 survivors after admission was evaluated in an Italian study and it was shown that serum 25-hydroxyvitamin (25-OHD) levels were lower in the long COVID group, in which patients with neurocognitive symptoms showed significantly lower levels of serum 25-OHD [14]. In another study conducted in Germany in which 8300 COVID-19 patients with vitamin D deficiency (VDD) were investigated, it was shown that patients with VDD had higher risks of post-acute emergency room visits, hospitalization and death, whereas an increased risk of the occurrence of long COVID was not detected in the VDD patients [15].

These clinical reports have implied that a low level of vitamin D is involved in the severity and prognosis of COVID-19; however, the impact of a low level of vitamin D on the manifestation of long COVID has yet to be determined, especially in Japanese patients. Therefore, in the present study, we attempted to elucidate the key symptoms and background characteristics related to the lowered vitamin D levels in Japanese patients with long COVID.

## 2. Patients and Methods

### 2.1. Patients’ Characteristics

The present study is a retrospective observational study conducted in our university hospital. On 15 February 2021, a COVID-19 aftercare outpatient clinic (CAC) was established in the Department of General Medicine, Okayama University Hospital [16]. We have been evaluating and treating patients suffering from long COVID symptoms at the CAC outpatient clinic. We define long COVID as symptoms that persist for more than four weeks after the onset of COVID-19 [17]. The symptoms associated with long COVID were identified by clinician diagnosis through face-to-face interviews with each physician. The patients who visited our CAC outpatient clinic from 27 May 2024 to 30 November 2024 were studied based on their medical records as of 17 December 2024. In accordance with our earlier report [18], the period since the first appearance of the Omicron variant in the Okayama district on 1 January 2022 was classified as the “Omicron-dominant period” of COVID-19, and only patients affected during the Omicron-dominant phase were included in the present study. In this study, long COVID patients more than ten years of age who visited the CAC during the study period were included. Patients’ information regarding age, gender, body mass index (BMI), current smoking habit, severity of COVID-19 in the acute phase [19], duration from the onset of COVID-19 to the first visit to the CAC, history of COVID-19 vaccination and clinical symptoms of long COVID were acquired from the medical records.

### 2.2. Inclusion and Exclusion Criteria

Data for patients who visited our clinic during the study period were obtained from their medical records. Of the 126 patients enrolled, we excluded 3 patients who were already taking vitamin D preparations, 21 patients who did not have blood tests for serum 25-OHD, 2 patients who developed the disease outside the Omicron variant period and 3 patients who did not meet diagnostic criteria. The remaining 97 patients with long COVID were taken for the clinical analysis related to vitamin D in the present study. Regarding the analysis of the timing of vitamin D measurement, 6 cases lacking the detailed date of initial infection were excluded. As for the laboratory data including corrected calcium, inorganic phosphate and alkaline phosphatase (ALP), 1 patient who did not have a blood test for serum ALP examination was excluded; 1 patient who did not undertake a blood test for serum zinc examination and 4 patients who were taking zinc preparations were also excluded from the 97 patients.

### 2.3. Determination of Vitamin D Deficiency

Serum 25-OHD concentrations were determined using Elecsys vitamin D total II reagent (Roche Diagnostics, Tokyo, Japan) by an automated analyzer in our clinical laboratory. In Japan, a deficiency of serum vitamin D is evaluated according to the “Guidelines for Determining Vitamin D Deficiency” established by the Japan Endocrine Society [20]. According to the guidelines, serum 25-OHD levels of 30 ng/mL or higher are defined as sufficient vitamin D, while serum 25-OHD levels less than 30 ng/mL, but not less than 20 ng/mL, are defined as insufficient vitamin D [20]. In this study, a serum 25-OHD concentration of less than 20 ng/mL was defined as VDD and a serum level of 20 ng/mL or higher was defined as non-deficient vitamin D (NDD).

### 2.4. Assessment of Fatigue State and Mental Condition

We evaluated patients’ fatigue status and mental status by having them complete a questionnaire using the Japanese version of the Fatigue Assessment Scale (FAS) [21] during their first outpatient visit. As for the FAS scoring in the patients with long COVID, we have confirmed the reliability and validity of the Japanese version of the FAS [22], showing that the Japanese version of the FAS is useful for assessing overall fatigue in Japanese patients with long COVID. The SDS (self-rating depression scale) questionnaire was used to assess mental health status including depression [23].

### 2.5. Statistical Analysis

All statistical analyses were performed using Stata/SE18. Pearson’s χ^2^ tests were used to compare the categorical variables. Linear regression analysis and Spearman’s rank correlation coefficients were used to test the interrelationships between the parameters. The Mann–Whitney U test and Student’s *t*-test were used for continuous variables with non-normal and normal distributions, respectively. Logistic regression analysis was performed for multivariate analysis and calculating odds ratios (ORs) and 95% confidence intervals (CIs). *p*-values of less than 0.05 were considered statistically significant.

### 2.6. Ethical Approval

This study was conducted with the approval of the Okayama University Hospital Ethics Committee (No. 2105-030) and complied with the Declaration of Helsinki. We posted information about the study on our website, and we offered patients who wished to opt out the opportunity to do so. Because the data were anonymized, informed consent from the patients was not required.

## 3. Results

**Figure 1:**
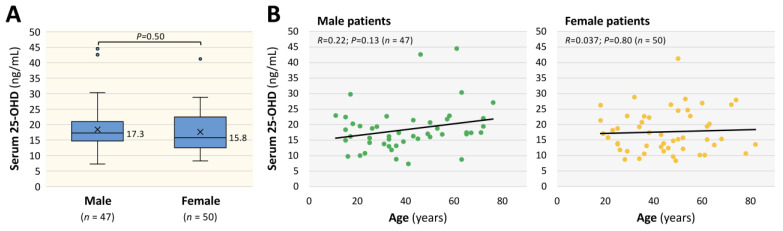
Serum 25-hydroxyvitamin D (25-OHD) concentrations in long COVID patients. (**A**) Association between serum 25-OHD concentrations (ng/mL) in male and female patients with long COVID. The boxes indicate the quartile range, the middle horizontal bar indicates the median, “x” indicates the mean and the outer lower and upper horizontal bars indicate the minimum and maximum values within 1.5 times the quartile range, respectively. Data for 25-OHD were analyzed by the Mann–Whitney U test. (**B**) The interrelationships between serum 25-OHD concentration (ng/mL) and patients’ age are shown as scatter diagrams for male patients (*n* = 47) and female patients (*n* = 50).

Of the 126 patients who visited our CAC during the study period, 97 patients with long COVID (male: 47; female: 50) were included in the analysis. As shown in Figure 1A, median concentrations of serum 25-OHD were 17.3 ng/mL in male patients and 15.8 ng/mL in female patients, and the difference was not significant. Also, as shown in Figure 1B, age-dependent changes in serum 25-OHD were not detected in male or female patients (male: R = 0.22, *p* = 0.13; *n* = 47 vs. female: R = 0.037, *p* = 0.80; *n* = 50).

**Figure 2:**
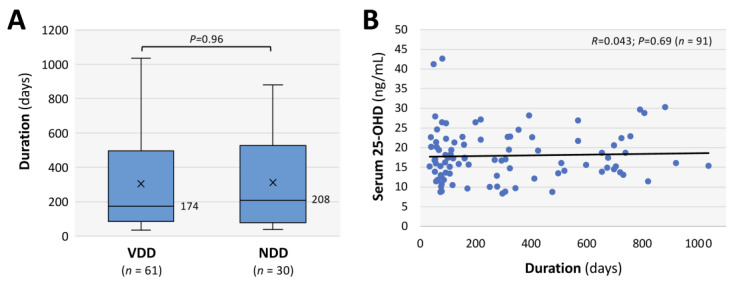
Duration from the initial infection to the measurements of serum 25-hydroxyvitamin D (25-OHD) concentrations in long COVID patients. (**A**) Durations in the groups with vitamin D deficiency (VDD: 25-OHD < 20 ng/mL) and non-deficient vitamin D (NDD: 25-OHD ≥ 20 ng/mL). The boxes indicate the quartile range, the middle horizontal bar indicates the median, “x” indicates the mean and the outer lower and upper horizontal bars indicate the minimum and maximum values within 1.5 times the quartile range, respectively. Data were analyzed by the Mann–Whitney U test. (**B**) The interrelationship between serum 25-OHD concentration (ng/mL) and the duration (days) is shown in the scatter diagram (*n* = 91).

The background characteristics of the long COVID patients in the VDD group (serum vitamin D concentration < 20 ng/mL) and the NDD group (serum vitamin D concentration ≥ 20 ng/mL) are shown in Table 1. A deficiency of serum vitamin D was detected in 66 (68%) of the 97 long COVID patients, whereas there were only 4 long COVID patients (4.1%) who had levels of serum 25-OHD above 30 ng/mL. The median concentrations of serum 25-OHD were 14.8 ng/mL and 22.9 ng/mL in the VDD group and the NDD group, respectively. The median ages of the patients were 38.5 years (interquartile range [IQR]: 26–52 years) in the VDD group and 50 years (IQR: 32–60 years) in the NDD group, and the VDD group included of 33 males (50%) and 33 females (50%). Other parameters including BMI, percentage of patients with a smoking habit, severity of COVID-19 in the acute phase, duration after the onset of COVID-19 to the first visit to the CAC, and vaccination conditions were not different between the two groups. The interrelationships between the durations from the initial infection to the measurement of serum 25-OHD and the serum levels of 25-OHD were also evaluated. As shown in Figure 2A, there were no significant differences in the durations between the VDD group and NDD group (median: 174 and 208 days, respectively). In addition, there were no significant interrelationship between the duration of follow-up period and serum levels of 25-OHD (*R* = 0.043; *p* = 0.69; *n* = 91; Figure 2B). Thus, no time-dependent changes of serum vitamin D levels were shown in the cases of long COVID. Moreover, serum levels of the biomarkers for bone metabolism, including serum corrected calcium, inorganic phosphate and ALP, were not significantly different between the VDD group and the NDD group (Table 2).

**Figure 3:**
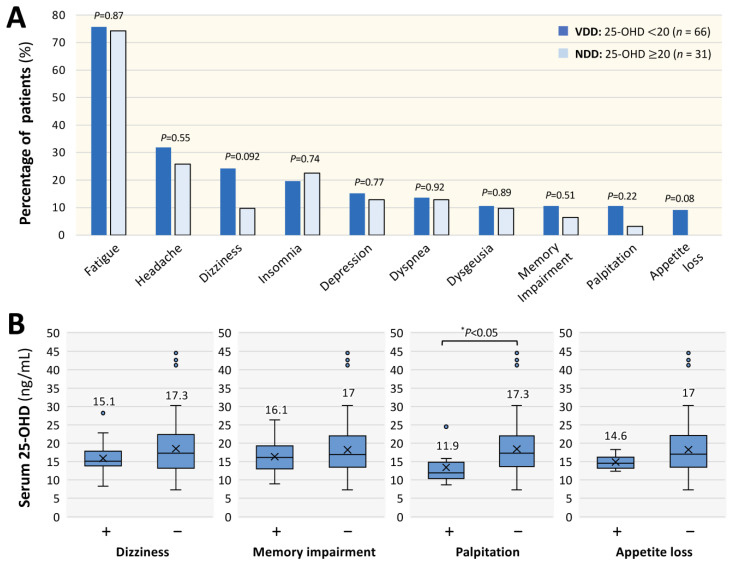
Symptomatic trends of long COVID patients with vitamin D deficiency and serum concentrations of 25-hydroxyvitamin D (25-OHD). (**A**) Percentages of patients who complained of major long COVID symptoms in the groups with vitamin D deficiency (VDD: 25-OHD < 20 ng/mL) and non-deficient vitamin D (NDD: 25-OHD ≥ 20 ng/mL). The chi-square test was performed. “*n*” denotes the number of patients. (**B**) Serum 25-OHD concentrations were compared between patients with and those without each symptom. The box indicates the quartile range, the middle horizontal bar indicates the median, “x” indicates the mean and the outer lower and upper horizontal bars indicate the minimum and maximum values within 1.5 times the quartile range, respectively. Data for 25-OHD were analyzed by the Mann–Whitney U test; statistical significance between the indicated groups is shown as * *p* < 0.05.

As for the differences in symptoms related to long COVID between the VDD group (*n* = 66) and the NDD group (*n* = 31), fatigue was the most common symptom in both groups (Figure 3A). Of note, the percentages of patients in the VDD group who complained of dizziness, memory impairment, palpitation and appetite loss were higher than the percentages of patients in the NDD group. As shown in Table 3, a logistic regression analysis of these major symptoms revealed that there were no symptoms with significantly higher OR for the VDD condition. However, the representative symptoms of long COVID, including dizziness (OR: 3.23) and palpitation (5.22), tended to have higher ORs, although statistically significant differences were not detected (Table 3). Nevertheless, as shown in Figure 3B, it was found that the patients who complained of palpitation had a significantly lower median concentration of serum 25-OHD than that in the patients without palpitation (11.9 vs. 17.3 ng/mL). The serum 25-OHD concentrations in long COVID patients with and in those without each symptom were not significantly different, as follows: dizziness (15.1 vs. 17.3 ng/mL), memory impairment (16.1 vs. 17 ng/mL) and decreased appetite (14.6 vs. 17 mg/mL; Figure 3B).

**Figure 4:**
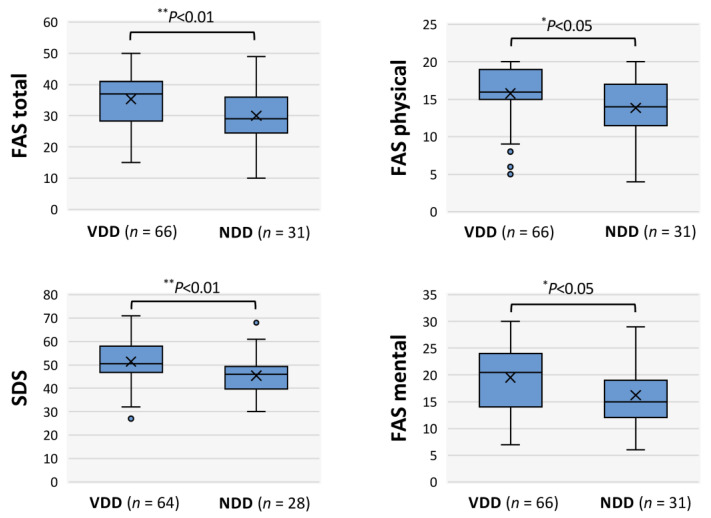
Scores of self-assessed scales for fatigue and depression in long COVID patients with vitamin D deficiency. FAS: Fatigue Assessment Scale; SDS: self-rating depression scale. VDD: vitamin D deficiency with serum concentration of 25-hydroxyvitamin (25-OHD) < 20 ng/mL; NDD: non-deficient vitamin D. The box is the interquartile range, the horizontal bar in the middle indicates the median, “x” indicates the mean and the lower and upper horizontal bars outside show the minimum and maximum values within 1.5 times the interquartile ranges, respectively. Data for FAS physical and FAS mental were analyzed by the Mann–Whitney U test and FAS total and SDS were analyzed by Student’s *t*-test; statistical significance between the indicated groups is shown as ** *p* < 0.01 and * *p* < 0.05. “*n*” denotes the number of patients.

Next, the self-rating scales for “fatigue” and “depression” were compared in the VDD and NDD groups, as shown in Figure 4. The scale scores (medians) for fatigue (FAS total, FAS physical, FAS mental) and depression (SDS) were significantly higher in the VDD group than in the NDD group: FAS total (37 vs. 29), FAS physical (16 vs. 14), FAS mental (20.5 vs. 15) and SDS (50.5 vs. 46; Figure 4). Thus, the VDD group had significantly higher FAS scores regarding physical and mental fatigue as well as higher SDS scores regarding depressive symptoms.

**Figure 5:**
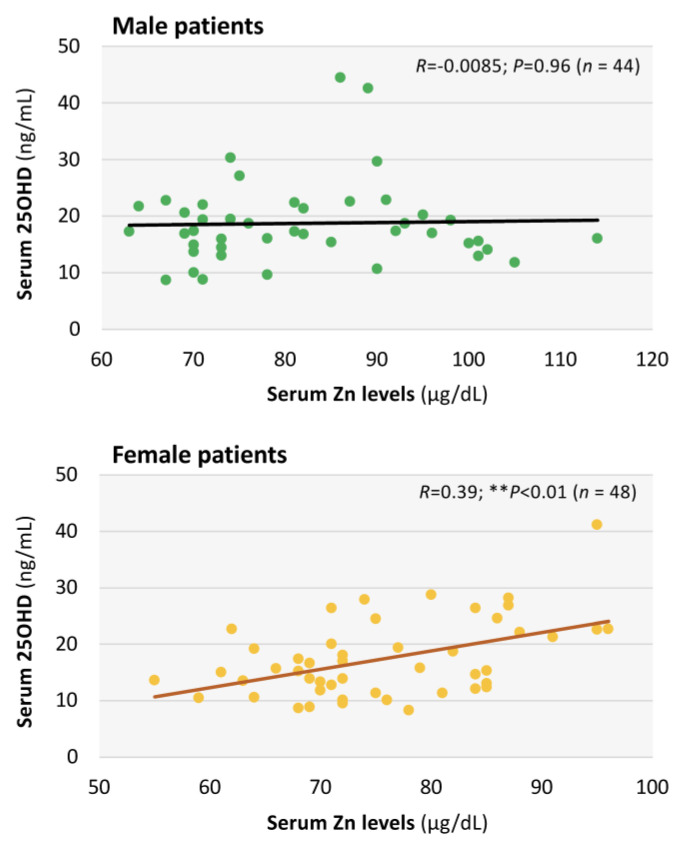
Interrelationships between serum concentrations of 25-hydroxyvitamin D (25-OHD) and zinc concentrations in long COVID patients. Interrelationships between serum levels of 25-OHD (ng/mL) and serum zinc concentrations (μg/dL) are shown as scatter diagrams in male patients (*n* = 44) and female patients (*n* = 48) with long COVID. ** *p* < 0.01 indicates a statistically significant difference.

Furthermore, as shown in Figure 5, an interrelationship between serum levels of 25-OHD and zinc was found in female patients (*R* = 0.39; *p* < 0.01; *n* = 48) but not in male patients (*R* = −0.0085; *p* = 0.96; *n* = 44).

## 4. Discussion

The present study revealed the proportion of long COVID patients with VDD and the clinical characteristics of VDD in long COVID patients. In the 97 patients included in this study, 66 patients (68%) with serum concentrations of 25-hydroxyvitamin D lower than 20 ng/mL were classified as the VDD group. The VDD group showed no specific background characteristics, including age, gender, BMI, severity and duration of COVID-19 and vaccination history. However, among the long COVID symptoms, large percentages of patients in the VDD group complained of dizziness, memory impairment, palpitation and appetite loss, and the patients who complained of palpitation had markedly low concentrations of serum 25-OHD (median: 11.9 ng/mL). Furthermore, patients in the VDD group had significantly higher FAS scores regarding physical and mental fatigue as well as higher SDS scores for depressive symptoms.

In the general population, it has been reported that the prevalence of VDD (<30 nmol/L; 12 ng/mL) in active elderly individuals is less than 5% and that the prevalence is much higher, about 50%, in inactive elderly individuals, depending on the activities of daily living [24]. Risk factors for vitamin D insufficiency include an unbalanced diet, aging, hospitalization, obesity, malabsorption, renal and hepatic damage and taking medications that promote vitamin D metabolism [25]. A cohort study showed that rates of VDD are higher in women, smokers, residents of mountainous regions and during winter, when sunlight hours are relatively short during the year [26]. In patients with long COVID, fatigue symptoms substantially affect employment conditions [27], leading to a decreased frequency of going out, and this lifestyle can be an inducer of decreased serum vitamin D levels.

In the present study, it was of interest that serum vitamin D levels were decreased in patients who complained of palpitation. There have been several reports regarding the clinical association of VDD with such cardiac symptoms [28,29,30,31], and the etiological involvement of VDD in arrhythmia has been suggested. One study showed that atrial electromechanical delay, which is an indicator for atrial fibrillation, is increased in patients with VDD [31]. It has also been reported that vitamin D is a negative modulator of the renin–angiotensin–aldosterone system [32], oxidative stress, and inflammation [33], resulting in myocardial remodeling and an increased risk for the development of atrial fibrillation. An in vitro study using adult mouse ventricular cardiomyocytes showed that vitamin D via vitamin D receptors (VDR)/Akt signaling increases sodium currents, the downregulation of which is the origin of electrophysiological remodeling [34]. Of note, VDD might have caused some structural remodeling before arrhythmia was detectable [31]. Long COVID patients with VDD in the present study might have been prone to feeling palpitations related to these latent factors.

In the present study, there were no laboratory or symptomatic abnormalities related to a calcium–phosphate imbalance. Severe VDD may also lead to rickets and osteomalacia, manifested by hypocalcemia and hypophosphatemia. Symptoms of hypocalcemia usually include tetany and muscle spasms and, in severe cases, convulsions and disorientation, and systemic symptoms include weakness, fatigue and depression. It has been shown that VDD is involved in an increased risk of coronary disease, cardiac hypertrophy, cardiomyopathy and heart failure, and VDD is also associated with many arterial aneurysms, arterial diseases and hypertensive atherosclerosis [35]. The mechanisms by which these conditions develop are mainly related to concomitant hypocalcemia; however, some direct effects via VDR on endothelial function may also be involved.

Vitamin D has been shown to be linked to cardiovascular benefits, since it affects calcium homeostasis, supports myocardial contractility and reduces the risk of cardiac hypertrophy and atherosclerosis [25,36]. Recent reviews have further shown that supplementation of vitamin D ameliorates cardiovascular risk factors including increases in high-density lipoprotein and decreases in triglycerides and blood pressure [37]. Vitamin D may also decrease inflammatory cytokines and reduce the risk of serious outcomes such as obesity and heart failure in COVID-19 patients [35]. Given that individuals with COVID-19 showed lowered expression of the VDR in peripheral blood cells, especially in male patients [38], the decreased sensitivities of the VDR should also be considered for evaluation of serum vitamin D concentration in patients with COVID-19.

Moreover, the condition of VDD has been reported to be associated with neurodegenerative diseases such as Alzheimer’s disease and Parkinson’s disease [39] and some psychiatric disorders, including schizophrenia [40]. VDD has also been shown to be involved in psychological stress [41], anxiety and depression [42,43,44]. In this regard, serotonin, a hormone that controls emotions and mood, might be involved since it is produced under the influence of vitamin D activity [45]. The neurotransmitter serotonin is generated from tryptophan and is released to the nervous system, especially in the brain [46]. The rate-limiting step for serotonin synthesis is the initial hydroxylation of tryptophan to 5-hydroxytryptophan that is regulated by tryptophan hydroxylase (TPH), followed by decarboxylation to form serotonin, wherein the key enzyme TPH is activated by the effects of vitamin D [47]. Serotonergic dysregulation has been recognized as a key etiology for various psychiatric conditions, such as attention deficit hyperactivity disorder, depression and possibly in the conditions of long COVID. Recently, Wong et al. demonstrated that the molecule neurologically associated with development of long COVID was serotonin [48]. They found that serotonin depletion driven by COVID-19-induced inflammation could be caused by reduced absorption of tryptophan, leading to decreased vagal and/or hippocampal activity and cognitive impairment as seen in brain fog [48]. Hence, vitamin D is likely to be important for maintaining optimal health of the brain and nervous system, particularly in long COVID conditions. In this regard, a retrospective cohort study of pediatric patients with Bell’s palsy reported that vitamin supplementation had no significant effect [49]. On the other hand, another study reported that vitamin D3 supplementation promoted recovery of facial nerve function and myelination in an animal model [50]. Interventional studies should be conducted to determine the therapeutic effects of vitamin D supplementation on the nervous system.

Moreover, an interrelationship between serum levels of 25-OHD and zinc was found in female patients with long COVID. This finding indicates that a common nutrition problem including vitamin D and zinc could be involved in the VDD in females but not males with long COVID. Alternatively, the correlation between serum 25-OHD and zinc levels only in female patients may be linked to the effects of sex steroids. We previously reported that serum zinc concentration in long COVID patients showed an age-dependent decrease only in male patients with long COVID [13]. It was also reported that activation of 25-OHD, i.e., the ratio of 1,25-dihydroxyvitamin D/25-OHD, correlates negatively with age in males and positively with BMI in females, suggesting that sex differences and BMI are related to vitamin D activation [51]. Hence, sex differences and the nutrition change with aging might be involved in the differences in the interrelationship between vitamin D and zinc levels in long COVID patients.

The present study has several research limitations. First, this study was a retrospective observational study at a single center in Japan. A multicenter prospective study including more patients would be needed to prove a causal relationship between hypovitaminosis D and long COVID symptoms. Second, this study only included patients who visited the CAC, so detailed fluctuations in patient background and comorbidities were not pursued. Third, the exact time of blood sampling was not considered in this study. Also, we did not account for seasonality, which is a major confounding factor for vitamin D levels. Fourth, we could not follow up the detailed time-course changes in the patients’ symptoms and serum levels of vitamin D, since the duration from the initial infection to the hospital visits for CAC were varied and the examinations for serum 25-OHD were limited to once in each patient due to the health insurance coverage. Fifth, the statistical power in the analysis of palpitation and serum 25-OHD concentrations was 62%; in the analysis of FAS and SDS for the VDD and NDD groups it was 74% and 79%, respectively, suggesting that larger sample sizes would be needed to draw decisive conclusions about the present results.

## 5. Conclusions

In conclusion, VDD might be involved in some specific symptoms of long COVID such as palpitation, fatigue and depression in Japanese patients. Further large-scale prospective studies of the patient group are necessary to elucidate the vitamin D-related pathophysiology of long COVID and to clarify the efficacy of vitamin D supplementation for patients with long COVID.

## Figures and Tables

**Table 1:** Background information of long COVID patients.

	NDD Group (*n* = 31)	VDD Group (*n* = 66)	*p*-Value
Median serum 25-OHD (ng/mL) [IQR]	22.9 [22.0–27.9]	14.8 [11.8–17.0]	<0.01 ^(a)^**
Median age (years) [IQR]	50 [32–60]	38.5 [26–52]	0.29 ^(a)^
Gender, *n* (%)			
Male	14 (45.2)	33 (50.0)	0.66 ^(b)^
Female	17 (54.8)	33 (50.0)	
Median BMI [IQR]			
	22.0 [18.9–25.8]	23.2 [19.1–25.5]	0.78 ^(c)^
Smoking habit, *n* (%)			
Yes	9 (29.0)	17 (25.8)	0.73 ^(b)^
No	22 (71.0)	49 (74.2)	
Severity of COVID-19 in acute phase, *n* (%)			
Mild	28 (90.3)	65 (98.5)	0.059 ^(b)^
Moderate/Severe	3 (9.7)	1 (1.5)	
Duration from the onset of COVID-19 to the first visit (%)			
≤90 days	9 (29.0)	20 (30.3)	0.90 ^(b)^
>90 days	22 (71.0)	46 (69.7)	
Vaccine, *n* (%)			
0 to 1 time	4 (12.9)	14 (21.5)	0.31 ^(b)^
≥2 times	27 (87.1)	51 (78.5)	
Unknown		1	

VDD: vitamin D deficiency with serum concentration of 25-hydroxyvitamin (25-OHD) < 20 ng/mL; NDD: non-deficient vitamin D; BMI: body mass index, COVID-19: coronavirus disease 2019. Medians [IQR: interquartile ranges] and percentages (%) are shown. ^(a)^ Mann–Whitney U test, ^(b)^ χ^2^ test and ^(c)^ Student’s *t*-test were performed to compare the two groups. ** *p* < 0.01 indicates statistically significant difference.

**Table 2:** Vitamin D-related laboratory data for long COVID patients.

Laboratory Parameters Related to Vitamin D	NDD Group	VDD Group	*p*-Value
Electrolytes			
Ca (mg/dL)	9.6 [9.3–9.8] (*n* = 31)	9.5 [9.2–9.7] (*n* = 66)	0.19 ^(a)^
corrected Ca (mg/dL)	9.7 [9.3–9.8] (*n* = 31)	9.5 [9.2–9.7] (*n* = 66)	0.12 ^(a)^
iP (mg/dL)	3.7 [3.4–3.9] (*n* = 31)	3.5 [3.3–3.8] (*n* = 66)	0.14 ^(b)^
Bone formation markers			
ALP (U/L)	68.5 [57–84] (*n* = 30)	73 [60–86] (*n* = 66)	0.54 ^(b)^

VDD: vitamin D deficiency with serum concentration of 25-hydroxyvitamin (25-OHD) < 20 ng/mL; NDD: non-deficient vitamin D; Ca: calcium; iP: inorganic phosphate; ALP: alkaline phosphatase. Medians [IQR: interquartile ranges] are shown. ^(a)^ Student’s *t*-test and ^(b)^ Mann–Whitney U test were performed to compare the two groups.

**Table 3:** Symptoms related to vitamin D deficiency in the patients with long COVID.

Long COVID Symptoms	Odds Ratio	95% CI	*p*-Value
Fatigue	0.93	0.31–2.78	0.89
Headache	1.13	0.40–3.19	0.82
Dizziness	3.23	0.81–12.9	0.096
Insomnia	0.60	0.18–2.00	0.41
Depression	1.18	0.29–4.84	0.81
Dyspnea	0.57	0.12–2.75	0.49
Dysgeusia	1.07	0.23–4.95	0.93
Memory impairment	1.49	0.27–8.15	0.64
Palpitation	5.22	0.43–63.6	0.20

A logistic regression analysis was performed, and the odds ratios related to decreases in serum vitamin D levels were evaluated for each major symptom of long COVID. CI: confidence interval.

## Data Availability

Detailed data will be made available if requested to the corresponding author. The data are not publicly available due to ethical reasons.

## Abbreviations

Body mass index (BMI); coronavirus disease 2019 (COVID-19); COVID-19 aftercare clinic (CAC); fatigue assessment scale (FAS); 25-hydroxyvitamin D (25-OHD); non-deficient vitamin D (NDD); post-COVID-19 condition (PCC); self-rating depression scale (SDS); tryptophan hydroxylase (TPH); vitamin D deficiency (VDD); vitamin D receptor (VDR).
